# Mustard Blisters

**Published:** 1874-12

**Authors:** 


Advice as to Blisters.—If you want a mustard-plaster, and don't want a mustard-blister, please observe this bit of advice: Don't mix your mustard with the white of an egg—don't do it—for if you do, language will fail you, fury will seize you, and you will discover, upon examination, that that embryotic chicken mixed with a little mustard had fire enough for a full-grown game rooster. No, no; stir up the mustard with anything else—vinegar, alcohol, gunpowder, or even nitro-glycerine, but shun the white of an egg as a mad dog would a pail of water. Yes, we know we told a different story some time since. We believed it then—we thought it a valuable contribution to the pharmaceutical knowledge of the country, and, as such, we wanted our readers to have the benefit of it. They have, and we don't overstate the case at all when we observe that one or two of them consider it altogether too much of a benefit. Why, the fact is, that mustard and the white of an egg mixed together and left alone will generate heat enough to kindle a small fire. Indeed, we should not be surprised if a careful analysis of a white heat would discover that mustard and eggs were the principal constituent parts. A friend of ours felt the need of a mustard plaster, and we had just printed that if the mustard was wet up with the white of an egg, all the beneficial results of the application could be realized without the blistering. Of course, the new process was tried. The plaster applied was about the size of your hand. The portion of the person from which the cuticle was entirely removed might have been covered with a palm-leaf fan. The fact is, it spread wider, burned deeper, bit sharper, and altogether proved the most blistering compound ever concocted in that household. The first information which we published on this subject we found in an exchange. The second—which is not like unto it—was conveyed to us from an individual who had taken our first statement in confidence. The first, evidently, was an ebullition from the brain of some crazy doctor; the last is born of experience, and is worth remembering. —*Tennessee Pharmacal Gazette, October*, 1874.

